# Vaccine Therapies for Prostate Cancer: Current Status and Future Outlook

**DOI:** 10.3390/vaccines12121384

**Published:** 2024-12-09

**Authors:** Wenhao Zhou, Xiaojun Lu, Feng Tian, Qianming Luo, Weihang Zhou, Siyuan Yang, Wenxuan Li, Yongjun Yang, Minfeng Shi, Tie Zhou

**Affiliations:** 1 Department of Urology, Shanghai Fourth People’s Hospital, School of Medicine, Tongji University, Shanghai 200434, China; zwh01215@163.com (W.Z.); xiaojunlucn@163.com (X.L.); 2 Department of Urology, Shanghai Eighth People’s Hospital, Shanghai 200235, China; tianf_sh@hotmail.com; 3 School of Medicine, Tongji University, Shanghai 200092, China; 18786125073@139.com (Q.L.); zhouwei20051006@163.com (W.Z.); evol5434@163.com (S.Y.); 4 College of Clinical Medicine, Naval Medical University, Shanghai 200433, China; 17661158806@163.com (W.L.); y2188058850@163.com (Y.Y.); 5 Reproduction Center, Changhai Hospital, Naval Medical University, Shanghai 200433, China

**Keywords:** prostate cancer, cancer vaccines, immunotherapy, combination therapies

## Abstract

Prostate cancer is a prevalent cancer in elderly men, and immunotherapy has emerged as a promising treatment approach in recent years. The aim of immunotherapy is to stimulate the body’s immune system to target and destroy cancer cells. Cancer vaccines that are highly specific, safe, and capable of creating long-lasting immune responses are a key focus in cancer immunotherapy research. Despite progress in clinical trials showing positive results, the practical use of cancer vaccines still encounters various obstacles. The complexity of the immune microenvironment and variations in the immune systems of individual patients have hindered the progress of research on prostate cancer vaccines. This review examines the history and mechanisms of cancer vaccines, summarizes recent clinical research findings, and explores future directions in the development of prostate cancer vaccines.

## 1. Introduction

Prostate cancer (PCa) is the most prevalent malignancy affecting the male genitourinary system [1]. Androgen deprivation therapy (ADT) serves as the primary treatment modality for patients with locally advanced or metastatic prostate cancer [2]. However, a significant proportion of patients eventually develop castration-resistant prostate cancer (CRPC) or metastatic disease, with a 5-year survival rate of approximately 30% for those with metastatic CRPC (mCRPC) [3]. While newer therapeutic approaches such as second-generation anti-androgen therapy, chemotherapy, and radiotherapy have demonstrated some efficacy in slowing the progression of advanced prostate cancer, they do not offer a definitive cure, and patients often succumb to the disease [4].

**Figure 1:**
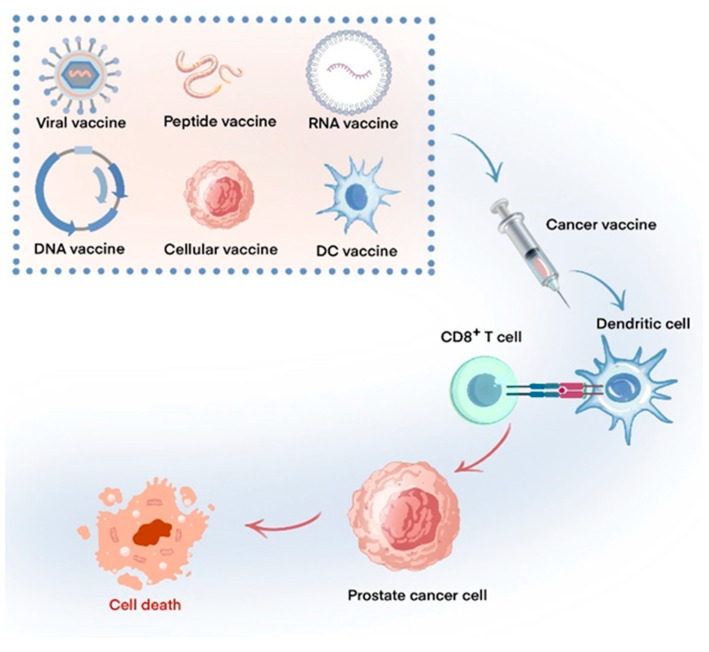
Different types of prostate cancer vaccines and their mechanisms. Prostate cancer vaccines can be categorized into several types, including peptide vaccines, viral vaccines, DNA-based vaccines, RNA-based vaccines, cellular vaccines, and dendritic cell-based vaccines. These vaccines are administered either intradermally or intramuscularly, allowing for the effective processing of tumor antigens by autologous antigen-presenting cells (APCs). The major histocompatibility complex (MHC) present on dendritic cells plays a crucial role in presenting antigens on their surface, which subsequently activates CD8+ T cells through binding to T cell receptors (TCRs), thereby enhancing the anti-tumor immune response.

Tumor immunotherapy, a treatment approach that leverages the body’s immune system to target and eliminate tumors, is considered a significant advancement in the realm of tumor therapy, often referred to as the third paradigm shift. This therapeutic strategy encompasses immune checkpoint inhibitors (ICIs) [5], adoptive cell therapy [6], and cancer vaccines [7] as its primary modalities. Cancer vaccines stimulate T cells within the patient’s system through the introduction of tumor antigens, leading to the initiation of an immune reaction aimed at eradicating tumor cells. The utilization of cancer vaccines has emerged as a favorable avenue within the realm of tumor immunotherapy owing to their notable specificity, favorable safety profile, and capacity to elicit enduring immune memory [8]. The progress in the development of cancer vaccines has been relatively sluggish in comparison to other forms of immunotherapy. Additionally, the significant heterogeneity observed within tumors presents considerable challenges in the creation of universal vaccines [9]. Prostate cancer vaccines include dendritic cell vaccines, viral vaccines, peptide vaccines, and DNA/mRNA vaccines, among others [10] (Figure 1). The FDA’s approval of Sipuleucel-T as the inaugural cancer vaccine for patients with mCRPC has underscored the potential significance of cancer vaccines in the management of prostate cancer [11]. Nonetheless, prostate cancer exhibits a pronounced vulnerability to immune evasion in response to immunotherapy, which can be attributed to its intrinsic characteristics, the immunosuppressive tumor microenvironment, and various biological and physiological factors related to the patient, including age and hormonal levels [12]. As a result, the efficacy of monotherapy utilizing immunotherapeutic agents is frequently limited. The adoption of combination therapy may provide improved treatment options for individuals diagnosed with prostate cancer [13].

Here, we examine the recent progress in utilizing vaccines for the treatment of prostate cancer, alongside exploring combination strategies that have undergone testing or are presently under assessment in clinical trials.

## 2. Dendritic Cell Vaccines

PROVENGE (Sipuleucel-T) is an autoimmune tumor vaccine that involves the ex vivo activation of isolated peripheral blood dendritic cells using a fusion protein composed of prostate acid phosphatase (PAP) and granulocyte–macrophage colony-stimulating factor (GM-CSF) [14]. This process aims to stimulate the generation of CD4+ and CD8+ T cells, thereby eliciting an immune response that contributes to anti-tumor effects [15]. Sipuleucel-T received marketing approval from the U.S. Food and Drug Administration (FDA) in 2010 and has shown both efficacy and safety in clinical trials. The findings from a substantial multicenter phase III clinical trial indicated that Sipuleucel-T therapy was associated with a 22% reduction in the risk of mortality and an extension of overall survival (OS) in patients with metastatic castration-resistant prostate cancer when compared to placebo (hazard ratio [HR] = 0.78, *p* = 0.03; median OS: 25.8 months versus 21.7 months) [16]. However, the therapy did not demonstrate a statistically significant improvement in progression-free survival (PFS), with results showing 3.7 months for the treatment group compared to 3.6 months for the placebo group (*p* = 0.63) [16]. In a comprehensive real-world study involving 6044 patients diagnosed with metastatic castration-resistant prostate cancer, the prognostic outcomes of Sipuleucel-T in conjunction with second-generation anti-androgen therapy (906 patients) were compared to those receiving second-generation anti-androgen therapy alone (5092 patients). The findings indicated that the combination treatment significantly extended overall survival for patients, with a median OS of 35.2 months for the combination group compared to 20.7 months for the monotherapy group (*p* < 0.0001) [17]. Prostate-specific antigen (PSA) levels were identified as a significant predictor of the treatment efficacy of Sipuleucel-T (*p* < 0.0001), with the treatment effect being more pronounced as the baseline PSA levels decreased [18]. The overall survival risk ratio for patients with a baseline PSA of ≤22.1 ng/mL was calculated to be 0.51 (95% confidence interval: 0.31 to 0.85), in contrast to a risk ratio of 0.84 (95% confidence interval: 0.55 to 1.29) for patients with PSA levels exceeding 134 ng/mL. These findings indicate that the administration of Sipuleucel-T is more advantageous in the earlier stages of cancer. The reduced tumor immunosuppression and concomitant increased activation of antigen-presenting cells (APCs) associated with early treatment are likely to enhance the ability of Sipuleucel-T to elicit long-term immune responses, thereby contributing to sustained clinical benefits.

Prostate cancer is characterized by a low level of immunogenicity and immune activity within an immunosuppressive tumor microenvironment, which is further exacerbated by a reduced presence of tumor-infiltrating CD8+ T cells [12,19]. Consequently, the response to immunotherapy in this context is markedly limited, leading to the predominant use of immunotherapy in conjunction with other treatment modalities. Theoretically, the administration of Sipuleucel-T is expected to activate and enhance immune responses, and its demonstrated long-lasting efficacy and safety suggest the potential for extended anticancer effects. Therefore, the incorporation of Sipuleucel-T in combination therapies is of significant clinical importance. The STAND trial was a randomized phase II study that assessed the efficacy of various dosing sequences of Sipuleucel-T in conjunction with ADT in patients experiencing biochemical recurrence of metastatic high-risk prostate cancer [20]. The findings indicated that the safety profile of the different dosing sequences was favorable. Notably, the administration of Sipuleucel-T prior to ADT resulted in a significantly enhanced specific T cell response compared to ADT alone (*p* = 0.001), and it was also associated with a more robust anti-tumor immune response [20]. The randomized phase II trial conducted by Small et al. assessed the efficacy of Sipuleucel-T in conjunction with abiraterone for the treatment of mCRPC [21]. The findings indicated that the simultaneous administration of these therapies did not diminish or modify the immune effects of Sipuleucel-T, and the combination was found to be well tolerated by participants [21]. Radiotherapy has the potential to work in conjunction with immunotherapy to strengthen and augment the anti-tumor immune response [22]. Radiotherapy possesses the capability to reduce tumor burden and enhance the infiltration of T cells into the tumor microenvironment. This process can convert “cold” tumors into “hot” tumors, making them amenable to effective targeting through immunotherapeutic strategies. Nevertheless, in a randomized phase II trial investigating the combination of Sipuleucel-T and radiotherapy, the administration of radiation to a single metastatic site at doses of up to 30 Gy in patients with asymptomatic or minimally symptomatic mCRPC did not result in an enhancement of the immune response linked to Sipuleucel-T therapy [23]. The effectiveness of Sipuleucel-T in combination with radiotherapy was assessed in a separate randomized phase II trial that included 32 patients diagnosed with bone-metastatic CRPC [24]. Participants were administered either Sipuleucel-T alone or in conjunction with ^223^Ra, an FDA-approved treatment for prostate cancer that specifically targets bone metastases. The findings revealed that patients receiving the combination therapy demonstrated more favorable clinical outcomes, particularly regarding PSA reduction, and no unforeseen safety concerns were identified, suggesting the absence of synergistic toxicity [24]. The existing literature on combination chemotherapy is limited, with only a few studies conducted in this area.

In certain research investigations, Sipuleucel-T was administered in conjunction with additional immune-modulating agents. A recent randomized phase II trial investigated the efficacy of combining Sipuleucel-T with the homeostatic cytokine interleukin-7 (IL-7), studying results either with or without its addition. The results demonstrated a significant expansion of CD4+ and CD8+ T cells and enhanced immune responses in patients receiving the combination therapy, as evidenced by a reduction in PSA levels compared to those treated with Sipuleucel-T alone [25]. A trial involving Sipuleucel-T and ipilimumab indicates that the combination therapy yields modest clinical responses while not significantly affecting antigen-specific responses [26]. A phase Ib study indicates that the combination of atezolizumab and Sipuleucel-T exhibits a favorable safety profile and may provide enhanced benefits, irrespective of the sequence of administration [27].

At present, Sipuleucel-T is not extensively utilized, primarily due to its restricted clinical efficacy and the treatment-related expenses that are often prohibitive for many patients. Nevertheless, the immunological advantages exhibited by Sipuleucel-T when used in combination therapy provide a foundation for further large-scale clinical trials.

DCVAC/PCa is composed of autologous dendritic cells that are cultured ex vivo in the presence of poly(I:C) and LNCaP prostate cancer cells, which are then administered subcutaneously as a vaccine [28]. This methodology has been evaluated in multiple early-phase clinical trials, either as a standalone treatment or in conjunction with radiotherapy or chemotherapy. Phase I/II small-sample clinical trials indicate that DCVAC/PCa treatment demonstrates a favorable safety profile for prostate cancer and significantly prolongs the prostate-specific antigen doubling time [29,30]. The VIABLE study [31], a phase 3 randomized clinical trial characterized by a double-blind, parallel-group, placebo-controlled design, included a total of 1182 male participants diagnosed with metastatic castration-resistant prostate cancer. The primary objective of the trial was to assess the safety and efficacy of the therapeutic agent DCVAC/PCa in conjunction with docetaxel. The findings indicated that the combination of DCVAC/PCa with docetaxel did not result in an extension of overall survival (OS) for patients with mCRPC, with survival durations of 23.9 months compared to 24.3 months, yielding a hazard ratio of 1.04 (95% confidence interval [CI]: 0.90–1.21, *p* = 0.60) [31]. In light of the unfavorable outcomes observed in the phase III trial, the development of DCVAC/PCa will not continue.

## 3. Cellular Vaccines

A cancer cell vaccine represents a therapeutic approach wherein the patient-derived cancer cell lines are modified and subsequently reintroduced into the patient’s body to elicit an immune response aimed at targeting the cancerous cells. The GVAX/PCa vaccine is classified as a genetically modified cancer cell vaccine, utilizing the LNCaP and PC-3 cell lines to produce and secrete granulocyte–macrophage colony-stimulating factor (GM-CSF) [32,33]. In a phase I/II trial conducted at a single institution involving patients diagnosed with recurrent, castration-sensitive prostate cancer, the researchers observed a significant reduction in PSA levels in 16 out of 21 patients (76%; *p* < 0.001) at the 20-week interval subsequent to the initial administration of GVAX treatment [34]. Another phase I/II clinical trial demonstrated that the percentage of patients with mCRPC generating antibodies in response to GVAX was positively correlated with the administered dose [35]. In light of the findings, two randomized phase III clinical trials were initiated. The VITAL-1 trial involved the randomization of asymptomatic patients diagnosed with mCRPC who had not previously received chemotherapy. These patients were divided into two groups: one group received GVAX, while the other was administered a combination of docetaxel and prednisone. In contrast, the VITAL-2 trial targeted symptomatic mCRPC patients, who were also chemotherapy-naïve, and randomized them into two groups: one group received a combination of GVAX and docetaxel, while the other group was treated with docetaxel in conjunction with prednisone [36]. Unfortunately, the VITAL-2 trial was halted prematurely after initial analyses indicated a survival benefit for the docetaxel plus prednisone cohort, alongside an alarming number of fatalities within the GVAX group. Following this, the VITAL-1 trial was also discontinued ahead of schedule due to concerns regarding its potential ineffectiveness in enhancing survival outcomes [37]. GVAX was also assessed in conjunction with other therapeutic agents. In a clinical trial involving patients with newly diagnosed prostate cancer who received treatment prior to undergoing prostatectomy, GVAX was evaluated in combination with androgen deprivation therapy and cyclophosphamide [38]. In this clinical trial, a total of 29 patients were randomly assigned to receive androgen deprivation therapy with degarelix either as a standalone treatment or following a regimen of cyclophosphamide and GVAX administered two weeks prior to the initiation of degarelix. At the two-year mark, 69% of patients who received the combination of GVAX, cyclophosphamide, and degarelix were free from PSA recurrence, in contrast to 40% of patients who were treated solely with degarelix. This difference, however, did not reach statistical significance, and the *p* value was not reported [38].

## 4. Peptide Vaccines

Tumor peptide vaccines consist of chemically synthesized peptides derived from established or anticipated amino acid sequences of antigenic epitopes. These vaccines are designed to target specific tumor antigens or cell surface proteins, thereby augmenting the immune system’s capacity to identify and eliminate tumor cells, ultimately promoting an anti-tumor immune response [39]. In patients with metastatic castration-resistant prostate cancer, peptide vaccines have been shown to reduce PSA levels and to elicit both cellular and humoral immune responses [40]. A randomized, double-blind, placebo-controlled phase III trial investigating the efficacy of peptide vaccines in patients with CRPC who experienced progression following docetaxel chemotherapy revealed that peptide vaccines did not result in an extension of OS or PFS in this patient population. However, subgroup analyses indicated that patients in the peptide vaccine treatment group with a low baseline neutrophil proportion or a high baseline lymphocyte proportion exhibited a significantly longer OS compared to those in the placebo group within this context [41]. A phase II clinical trial investigating the use of peptide vaccines in conjunction with low-dose cyclophosphamide for the treatment of mCRPC demonstrated a reduction in PSA levels in a limited cohort of patients across both treatment groups. However, no significant differences were noted in OS, PFS, or antigen-specific T cell activity [42]. KRM-20 is an innovative tumor vaccine formulated with 20 peptides designed to stimulate cytotoxic T lymphocyte responses against 12 distinct tumor-associated antigens that are prominently expressed in prostate cancer tissues. A phase II clinical trial was conducted to evaluate the efficacy of KRM-20 in conjunction with docetaxel and dexamethasone in enhancing anti-tumor effects in patients diagnosed with CRPC. The results indicated no significant differences in OS and PFS between the experimental group and the placebo group. However, KRM-20 demonstrated effectiveness in patients with chronic prostate cancer characterized by lymphocyte levels of 26% or higher, or PSA levels below 11.2 ng/mL [43]. Recent advancements in bioinformatics have led to an increased interest in peptide vaccines that utilize individualized neoantigens within the realm of oncology. Nevertheless, the restricted selection of available adjuvants, coupled with the immunosuppressive microenvironment characteristic of tumors, continues to pose significant challenges in the treatment of cancer [44].

## 5. Nucleic Acid Vaccines

Tumor nucleic acid vaccines function by eliciting an immune response through the administration of DNA or RNA sequences that encode tumor antigens, thereby facilitating the destruction of tumor cells. The primary benefits of nucleic acid vaccines encompass their design flexibility, capacity to induce a wide range of immune responses, and favorable safety and stability profiles. Preliminary research has demonstrated their efficacy in enhancing immune responses, particularly in relation to tumor-specific CD8+ T cells [45].

### 5.1. DNA Vaccine

DNA vaccines consist of closed-loop DNA plasmids that are engineered with specific target antigens. These plasmids encode antigens that are regulated by a robust mammalian promoter, which serves to augment immunogenicity and facilitate the destruction of tumor cells via targeted antigen presentation [46]. Upon administration of the DNA vaccine into the host organism, the DNA contained within the vaccine is transcribed and translated within antigen-presenting cells (APCs) to generate specific tumor antigens. These antigens can subsequently be presented on major histocompatibility complex (MHC) class I and class II molecules through mechanisms such as direct presentation, secretion, or via apoptotic vesicles [47,48,49]. This process facilitates the activation of CD8+ T cells and CD4+ T cells, thereby eliciting targeted immune responses. Furthermore, as an exogenous entity, the plasmid DNA’s double-stranded configuration can activate intracellular nucleic acid receptor sensing signaling pathways, leading to the induction of intrinsic immune responses [50]. Additionally, the presence of CpG oligonucleotide sequences within the plasmid can stimulate the Toll-like receptor 9 (TLR9) signaling pathway, resulting in the production of chemokines and inflammatory mediators, including CXCL10 and IL-6, which enhance the immunogenic efficacy of the vaccine [51]. The predominant DNA-based vaccines for prostate cancer are primarily designed to encode proteins such as Prostatic Acid Phosphatase (PAP), Prostate-Specific Membrane Antigen (PSMA), Prostate-Specific Antigen (PSA), and Androgen Receptor (AR), among others [52].

The most extensively researched antigen in DNA vaccine trials for prostate cancer is PAP. A phase II clinical trial was conducted to evaluate the impact of a DNA vaccine encoding PAP (pTVG-HP) on tumor metastasis in patients with recurrent, non-metastatic prostate cancer [53]. A total of 99 patients with desmoplasia-sensitive PCa and a PSA doubling time (PSADT) of less than 12 months were randomly assigned to receive either pTVG-HP in conjunction with a 200 μg GM-CSF adjuvant administered intradermally, or 200 μg GM-CSF alone, with both treatments administered six times at 14-day intervals over a period of two years. The primary endpoint of the study was the two-year metastasis-free survival rate (MFS). The findings indicated that pTVG-HP did not result in a statistically significant improvement in the two-year MFS for patients with desmoplasia-sensitive PCa, with the MFS recorded at 42.7% for the vaccine group compared to 41.8% for the GM-CSF alone group. Consequently, further investigations utilizing pTVG-HP in combination with PD-1 inhibition are currently in progress [53]. Findings from a concurrent trial indicated that intensive treatment with pTVG-HP following Sipuleucel-T vaccination elicited a more robust PAP antibody response in patients with mCRPC. However, no significant differences were observed in disease progression or overall survival between the treatment groups [54]. A recent phase I/IIa clinical trial evaluating a vaccine targeting encoded PAP demonstrated a favorable safety profile [55]. Specifically, 14% (3 out of 22) of participants exhibited the production of PAP-specific IFN-γ-secreting CD8+ T cells immediately following treatment. Additionally, 41% (9 out of 22) of the patients displayed PAP-specific proliferation of CD4+ and/or CD8+ T cells. Notably, there was a statistically significant difference observed in the PSADT with a *p*-value of less than 0.05.

Prostate-specific antigen (PSA) is a well-established biomarker for prostate cancer and serves as the primary target for the investigation of DNA vaccines aimed at treating this malignancy. In a phase I dose-escalation trial [56], a plasmid containing the complete sequence of PSA was administered to patients with CRPC in monthly intervals over a period of 5 months. Notably, only the maximum dosage of 900 μg of DNA resulted in the generation of PSA-specific cellular and humoral antibody responses. To enhance the immunogenicity of this PSA vaccine, researchers conducted an investigation involving a DNA vaccine that encodes the Rhesus PSA gene [57]. This vaccine was delivered via electroporation to facilitate the transfection of antigen-presenting cells. A total of 15 patients were mandated to initiate androgen deprivation therapy prior to receiving the vaccination. Notably, all but one patient exhibited pre-existing PSA-specific T cells, the frequency of which was augmented either through androgen deprivation or vaccination. This phenomenon complicates the interpretation of the results obtained from this trial.

The androgen receptor serves as the principal pharmacological target in the treatment of prostate cancer. Another DNA-based vaccine, known as pTVG-AR, specifically targets the ligand-binding domain of the androgen receptor. Preclinical investigations conducted in murine models of prostate cancer have demonstrated the safety and anti-tumor efficacy of pTVG-AR [58]. Furthermore, a study involving patients with metastatic castration-sensitive prostate cancer undergoing ADT revealed that pTVG-AR elicited Th1-type anti-tumor immunity in 47% of the participants, which is associated with a significant extension of progression-free survival [59].

PSMA represents a compelling target for the development of anti-PSMA monoclonal antibodies aimed at both diagnostic and therapeutic applications due to its classification as a membrane protein [60]. The elevated expression of PSMA in the epithelial cells of prostate adenocarcinoma further enhances its potential as a target for vaccination strategies [61]. In a phase I/II dose-escalation trial, the efficacy of a DNA vaccine encoding fusion proteins, including PSMA and a T helper cell stimulator, was evaluated in patients diagnosed with prostate cancer. The findings revealed a significant increase in the frequency of CD8+ and CD4+ T cells among the participants. Notably, vaccinated individuals demonstrated a substantial enhancement in PSA doubling time, which improved from 11.97 months prior to treatment to 16.82 months following treatment (*p* = 0.04) when compared to the control group that did not receive the vaccine. Additionally, within the vaccinated population, there was a pronounced increase in PSMA-specific T cells post-vaccination in relation to baseline measurements [62]. A recent investigation demonstrated that the incorporation of a peptide derived from PSMA or T cell receptor γ alternate reading frame protein (TARP) into an optimized spherical nucleic acid (SNA) vaccine markedly influenced the adaptive immune response to established prostate cancer targets in clinical settings [63]. This finding suggests that the integration of SNA peptides into optimized SNA vaccines could represent a promising avenue for future research in the field of prostate cancer immunotherapy.

### 5.2. RNA Vaccine

mRNA vaccines are developed by introducing mRNA that encodes antigenic proteins into the organism, which are subsequently translated to produce these proteins, thereby eliciting an immune response. In comparison to vaccines that utilize DNA, mRNA vaccines offer several advantages, including the ability to express multiple antigens simultaneously, high efficiency of in vivo expression, non-infectious properties, non-integrated expression, and the absence of insertional mutations, all contributing to their safety profile [64,65]. Furthermore, mRNA vaccines are characterized by high production efficiency, cost-effectiveness, and ease of large-scale preparation. Consequently, mRNA-based tumor vaccines exhibit promising potential for future development [66,67].

The anti-tumor vaccine CV9103 represents the sole reported vaccine strategy utilizing mRNA via direct injection as of the present date. This vaccine comprises four self-adjuvant mRNAs that encode for PSA, six-transmembrane epithelial antigen of the prostate 1 (STEAP1), PSMA, and prostate stem cell antigen [68]. In a phase I/IIa clinical trial, CV9103 was administered via intradermal injection to a cohort of 44 patients diagnosed with CRPC. The study findings indicated that there was no significant correlation between immune response and overall survival. The median OS for the entire patient population was recorded at 29.3 months, while the median OS for the subgroup of patients with metastatic disease was slightly higher at 31.4 months [68]. CV9104 is a second-generation vaccine that utilizes sequence-optimized, free, and protamine-complexed mRNA to encode the antigens PSA, PSMA, PSCA, STEAP1, PAP, and MUC1. This vaccine was assessed in a randomized phase I/IIb clinical trial involving 197 patients with chemotherapy-naïve mCRPC. The results indicated that CV9104 did not demonstrate a statistically significant improvement in OS when compared to the placebo group (35.5 vs. 33.7 months, *p* = 0.33) [69]. It is anticipated that advancements in antigen screening methodologies, along with the integration of immune checkpoint inhibitors (ICIs) and other therapeutic approaches, will further augment the immunological activity and clinical efficacy of mRNA vaccines in the future.

## 6. Viral and Bacterial Vaccines

Viral vaccines employ modified viruses, such as adenoviruses or virus-like particles, as vectors to transport genetic material that encodes tumor antigens into host cells. This process prompts the host cells to express these antigens, thereby stimulating an immune response. The advantages of these vaccines include their effective delivery of tumor antigens, the induction of a robust immune response, and their specificity for tumor cells [70]. PROSTVAC (PSA-TRICOM) is a vaccine that utilizes a viral vector approach, which involves the incorporation of a recombinant plasmid containing a PSA transgene into a poxvirus. Additionally, this vaccine includes a plasmid that encodes three viral T cell costimulatory molecules, collectively referred to as TRICOM [71]. TRICOM is composed of B7.1, ICAM-1, and LFA-3, and it primarily functions to augment T cell affinity and promote the lysis of tumor cells [72]. Findings from a phase II clinical trial indicated that patients with mCRPC who were administered PROSTVAC experienced a median overall survival extension of 9.9 months (26.2 months compared to 16.3 months) [73]. A subsequent phase III clinical trial revealed that, although PROSTVAC treatment exhibited an acceptable safety profile and tolerability, it did not result in a statistically significant improvement in OS compared to placebo in patients with mCRPC, with median OS reported as 34.4 months for PROSTVAC and 34.3 months for placebo (*p* = 0.47) [74]. A phase II open-label study investigating PROSTVAC was conducted with 25 patients diagnosed with localized prostate cancer who had undergone radical prostatectomy. The findings revealed that 13 patients (52%) exhibited peripheral T cell responses to more than one of the three tumor-associated antigens (TAAs) assessed. Notably, five of these patients demonstrated responses to all three TAAs evaluated. These results indicate that PROSTVAC has the potential to elicit both tumor-specific immunity and peripheral immune responses [75]. Adenoviruses were initially employed as vectors for gene therapy. However, in recent years, their application as vaccine vectors has expanded, particularly with the advancement of next-generation vectors designed for genetically modified products that exhibit enhanced safety and immunogenicity [76]. Ad5-PSA is a tumor vaccine that utilizes a replication-defective recombinant adenovirus 5 (Ad5) vector. Recently, a novel vaccine has been developed that targets three TAAs, PSA, Brachyury, and MUC-1, employing the Ad5 carrier [E1-, E2b-]. A phase I clinical trial has demonstrated that this vaccine is capable of reducing PSA levels and stabilizing the disease in patients with mCRPC, exhibiting favorable tolerability and an acceptable safety profile [77].

The listeria monocytogenes (LM) vaccine, which is an attenuated formulation derived from LM, elicits an immune response via listeriolysin O (LLO) therapy. Initial findings from an animal study indicate that the administration of a DNA vaccine in conjunction with the LM vaccine resulted in a robust anti-tumor immune response mediated by CD4+ T cells [78]. In a phase I/II clinical trial involving 50 patients diagnosed with mCRPC, an attenuated strain of Listeria monocytogenes that encodes PSA, referred to as ADXS31-142, was administered. Participants received either ADXS31-142 as a monotherapy (*n* = 13) or in conjunction with pembrolizumab (*n* = 37). The study did not report any objective radiographic responses; however, one patient (13%) in the monotherapy group and five patients (17%) in the combination group exhibited a reduction in PSA levels of 50% or greater from baseline. Notably, the median overall survival was recorded at 7.5 months for the vaccine-only cohort, while it was significantly longer at 33.7 months for those receiving the combination therapy [79]. The investigation of various targets remains a significant concept in the pursuit of novel vaccine development. Table 1 presents a summary of clinical trials involving vaccines administered to patients diagnosed with prostate cancer.

## 7. Conclusions

Cancer vaccines represent a promising avenue for immunotherapeutic intervention in the prevention and treatment of tumors, as they have the potential to elicit tumor-antigen-specific immune responses and establish immunological memory, thereby offering significant clinical benefits. This review delineates the mechanisms underlying the action of prostate cancer vaccines and categorizes their classification and progress in clinical trials. Despite the extensive research conducted on prostate cancer vaccines over the years, only Sipuleucel-T has received approval for clinical application, while the majority of tumor vaccines remain in phase I/II clinical trials. Current efforts in prostate cancer immunotherapy primarily focus on investigating and refining novel immunotherapeutic strategies informed by comprehensive studies of the biological behavior of prostate cancer cells; examining the role of immune checkpoint inhibitors in elucidating mechanisms of immune evasion; and developing new immunotherapeutic agents that demonstrate enhanced efficacy with reduced adverse effects. Prostate cancer is characterized as a prototypical “cold tumor”, exhibiting low levels of T cell infiltration and other factors that impede immune function. Consequently, enhancing the immune sensitivity of prostate cancer is of paramount importance for effective treatment. Concurrently, advancements in sequencing technologies are facilitating the rapid analysis and synthesis of patient-specific tumor neoantigens, positioning neoantigen-based vaccines as a critical focus within the field. Furthermore, the intricate nature of the tumor immune microenvironment renders monotherapy insufficient, suggesting that combination therapies may represent the most effective strategy to address this challenge, thereby highlighting an essential direction for the application of cancer vaccines.

## Figures and Tables

**Table 1:** Clinical trials with vaccines in prostate cancer to date.

Vaccine Type	Vaccine	Combination	Disease	N	Trail Phase	Results or Comments	Clinical Trial ID
Dendritic cell vaccines	Sipuleucel-T	NA	mCRPC	512 (341 vs. 171)	III	The extended OS observed in patients administered the vaccine, in contrast to those receiving a placebo, resulted in the approval of the vaccine by the FDA in 2010 [16]	NCT00065442
	Sipuleucel-T	Androgen receptor signaling pathway inhibitors (ASPIs)	mCRPC	6044 (906 vs. 5092)	Retrospective	The administration of Sipuleucel-T at any point in time was correlated with enhanced OS when compared to the use of ASPI alone [17]	
	Sipuleucel-T	NA	Androgen-dependent prostate cancer	176 (117 vs. 59)	III	No significant difference in the time to biochemical failure was detected [80]	NCT00779402
	Sipuleucel-T	ADT	Biochemically recurrent prostate cancer	68 (34 vs. 34)	II	The administration of Sipuleucel-T prior to ADT resulted in a significantly enhanced specific T cell response compared to ADT alone [20]	NCT01431391
	Sipuleucel-T	Abiraterone	mCRPC	69 (35 vs. 34)	II	The combination of Sipuleucel-T and abiraterone was found to be well tolerated, with no new safety concerns identified [21]	NCT01487863
	Sipuleucel-T	Radiation therapy	mCRPC	51 (24 vs. 25)	II	Radiation therapy did not improve the humoral and cellular immune responses linked to Sipuleucel-T treatment [23]	NCT01807065
	Sipuleucel-T	IL-7	mCRPC	56 (26 vs. 28)	II	Augmented immune responses observed in patients undergoing combination therapy [25]	NCT01881867
	Sipuleucel-T	Ipilimumab	mCRPC	50 (26 vs. 24)	II	The combination of ipilimumab and Sipuleucel-T demonstrated limited clinical efficacy and did not significantly modify antigen-specific responses [26]	NCT01804465
	DCVAC/PCa	Docetaxel and prednisone	mCRPC	1182 (787 vs. 395)	III	No improvement in OS of patients with mCRPC [31]	NCT02111577
Cellular vaccines	GVAX	NA	mCRPC	600	III	Terminated [36]	NA
	GVAX	Degarelix	Localized prostate cancer	29 (15 vs. 14)	NA	GVAX demonstrated a modest enhancement of the immunological effects associated with ADT [38]	NA
Peptide vaccines	Personalized peptide vaccines	NA	CRPC	310 (207 vs. 103)	III	No improvement in OS [41]	UMIN000011308
	Personalized peptide vaccines	Cyclophosphamide	mCRPC	310 (35 vs. 35)	II	No improvement in OS and PFS [42]	UMIN000005329
	KRM-20	Docetaxel and dexamethasone	CRPC	51 (25 vs. 26)	II	No improvement in OS and PFS [43]	UMIN000011028
DNA vaccines	pTVG-HP	NA	nmCSPC	99 (48 vs. 49)	II	Two-year MFS was not different overall between study arms [53]	NCT01341652
	pTVG-HP	sipuleucel-T	mCRPC	18 (9 vs. 9)	II	Median time to progression was not significantly different [54]	NCT01706458
	pVAX/PSA	NA	CRPC	8	I	At maximum dose (900 µg), PSA-specific cellular and humoral immunity detected [56]	NA
	pTVG-AR	NA	mCSPC	40	I	Patients exhibiting T cell immunity demonstrated a marked extension of PFS [59]	NCT02411786
	pDOM-PSMA_27_	NA	Biochemically recurrent prostate cancer	64 (32 vs. 32)	I/II	A notable improvement in PSA doubling time was observed among patients who received the vaccine [62]	NA
RNA vaccines	CV9103	NA	CRPC	44	I/IIa	There was no significant correlation between immune response and OS [68]	2008-003967-37
Viral and bacterial vaccines	PROSTVAC	GM-CSF	mCRPC	1297 (429 vs. 429 vs. 428)	III	PROSTVAC demonstrated a favorable safety profile and was well tolerated; however, no significant enhancement in overall survival rates was observed [74]	NCT01322490
	Ad5-PSA	NA	mCRPC	18	I	This vaccine demonstrates a favorable tolerability and an acceptable safety profile [77]	NCT03481816
	ADXS31-142	pembrolizumab	mCRPC	50 (37 vs. 13)	I/II	The combination of ADXS31-142 with pembrolizumab has been shown to enhance the median OS [79]	NCT02325557
